# Chromosome-level genome assembly of bean flower thrips *Megalurothrips usitatus* (Thysanoptera: Thripidae)

**DOI:** 10.1038/s41597-023-02164-5

**Published:** 2023-05-03

**Authors:** Ling Ma, Qiaoqiao Liu, Shujun Wei, Shanlin Liu, Li Tian, Fan Song, Yuange Duan, Wanzhi Cai, Hu Li

**Affiliations:** 1 Department of Entomology and MOA Key Lab of Pest Monitoring and Green Management, College of Plant Protection, China Agricultural University, Beijing, 100193 China; 2 Sanya Institute of China Agricultural University, Sanya, 572025 China; 3 Institute of Plant Protection, Beijing Academy of Agriculture and Forestry Sciences, Beijing, 100097 China

**Keywords:** Genomics, Entomology

## Abstract

Bean flower thrips *Megalurothrips usitatus* is a staple pest of cowpea and other legumes and causes dramatic economic losses. Its small size allows for easy concealment, and large reproductive capacity easily leads to infestations. Despite the importance of a genome in developing novel management strategies, genetic studies on *M. usitatus* remain limited. Thus, we generated a chromosome-level *M. usitatus* genome using a combination of PacBio long read and Hi-C technologies. The assembled genome was 238.14 Mb with a scaffold N50 of 13.85 Mb. The final genome was anchored into 16 pseudo-chromosomes containing 14,000 genes, of which 91.74% were functionally annotated. Comparative genomic analyses revealed that expanded gene families were enriched in fatty acid metabolism and detoxification metabolism (ABC transporters), and contracted gene families were strongly associated with chitin-based cuticle development and sensory perception of taste. In conclusion, this high-quality genome provides an invaluable resource for us to understand the thrips’ ecology and genetics, contributing to pest management.

## Background & Summary

Bean flower thrips *Megalurothrips usitatus* is a highly harmful pest of leguminous crops in the genera *Glycine*, *Arachis*, and *Vigna*^1–4^. The insect lays eggs in plant tissue and feeds on leaves, flowers and pods, causing economic losses worldwide, particularly in southern China, India, Japan, the Philippines, and Australia^1,3,5,6^. Its small body size, cryptic behavior, and fast transmission present difficulties in pest control^6,7^.

Attempts to mitigate agricultural damage have largely involved chemical insecticides^8–12^. However, excessive pesticide usage leaves residues that risk consumer health and also induce resistance in pest insects. Understanding the evolution of pesticide resistance is necessary for developing novel management strategies, but the genetics of *M. usitatus* remains poorly understood. Filling this knowledge gap will benefit our efforts at pest control.

In this study, we assembled a chromosome-level genome of *M. usitatus* using a combination of PacBio long read, Illumina short-read sequencing, and chromosome conformation capture (Hi-C) technologies. We compared the genomic features of *M. usitatus* with those of other insects to explore the genomic signatures of resistance. The high-quality reference genome of the bean flower thrips obtained in this study will lay the foundation for future investigations on the ecology of thrips and provide valuable genetic information for its management.

## Methods

### Sample preparation and genomic DNA sequencing

*Megalurothrips usitatus* samples were collected from Wanning, Hainan province, and reared for approximately 100 generations in the laboratory. Adults were fed *Lablab purpureus* and kept at 25 ± 1 °C, 70 ± 5% relative humidity, and 14:10 light:dark cycle. Stages were confirmed under a light microscope and verified with pictorial keys^13^. Individuals were then quickly placed into collection tubes, flash-frozen in liquid nitrogen, and stored at −80 °C until use.

We prepared approximately 2,000 mixed-sex *M. usitatus* individuals for genome sequencing. Genomic DNA was extracted using the CTAB method, followed by purification using a Blood and Cell Culture DNA Midi Kit (QIAGEN, Germany). The purity and concentration of extracted DNA were determined with 0.75% agarose gel electrophoresis and a Qubit 2.0 Fluorometer (Thermo Fisher Scientific, USA), respectively. The library constructed from the extracted DNA was approximately 10–20 Kb in size. A PacBio Sequel sequencer (Pacific Biosciences, Menlo Park, USA) was used for long DNA fragments, and Illumina Novoseq 6000 was used to generate 150 bp paired-end short reads. The sequencing yielded 98.30 Gb (412.78 × coverage) of long-reads with an N50 length of 14,475 bp and an average length of 10,352.68 ± 2.46 bp (mean ± S.E.). The Illumina platform sequenced 58.80 Gb raw data, of which adapters and low-quality short reads were removed using Fastp version 0.21.0^14^ with default parameters, resulting in a total of 55.86 Gb (234.57 × coverage) clean data (Table 1).

**Table 1:** Library sequencing data and methods used in this study to assemble the *Megalurothrips usitatus* genome.

Sequencing strategy	Platform	Usage	Insertion size	Clean data (Gb)	Coverage (X)
Short-reads	Illumina	Genome survey	350 bp	55.86	234.57
Long-reads	PacBio-sequel II	Genome assembly	10–20 Kb	98.30	412.78
Hi-C	Illumina	Hi-C assembly	350 bp	53.90	226.34
RNA-seq	Illumina	Anno-evidence	350 bp	5.61	23.56
Full-length transcriptome	PacBio-sequel II	Anno-evidence	1–10 Kb	47.67	200.18

### Hi-C library preparation and sequencing

Chromosome conformation capture (Hi-C) sequencing used fresh tissues from 1,500 mixed-sex *M. usitatus* individuals. The samples were cross-linked with a 2% formaldehyde isolation buffer and then treated with DpnII (NEB) to digest nuclei. Biotinylated nucleotides were used to repair the tails, and the ligated DNA was split into fragments of 350 bp in length. The resulting Hi-C library was sequenced in Illumina Novoseq. 6000 with paired-end 150 bp. After applying the same filter criteria for short reads, a total of 53.90 Gb (226.34 × coverage) of clean data was generated (Table 1).

### Transcriptome sequencing

A pooled *M. usitatus* sample was prepared using 30 eggs, 20 pseudo-pupae, 10 females, and 10 males. Total RNA was extracted using the TRIzol reagent (Thermo Fisher Scientific, USA). A paired-end library was constructed using the TruSeq RNA Library Preparation Kit (Illumina, USA) and sequenced on an Illumina Novoseq 6000 platform. It resulted in a total of 5.61 Gb RNA-seq clean data (Table 1). Additionally, total RNA (1 µg) was used to construct a full-length transcript isoform library using the SMRT bell Express Template Prep Kit 2.0 (Pacific Biosciences, USA). Target-size sequences were generated using the PacBio sequel II platform. A total of 47.67 Gb full-length transcriptome data was obtained (Table 1).

### Estimation of genomic characteristics

**Fig. 1:**
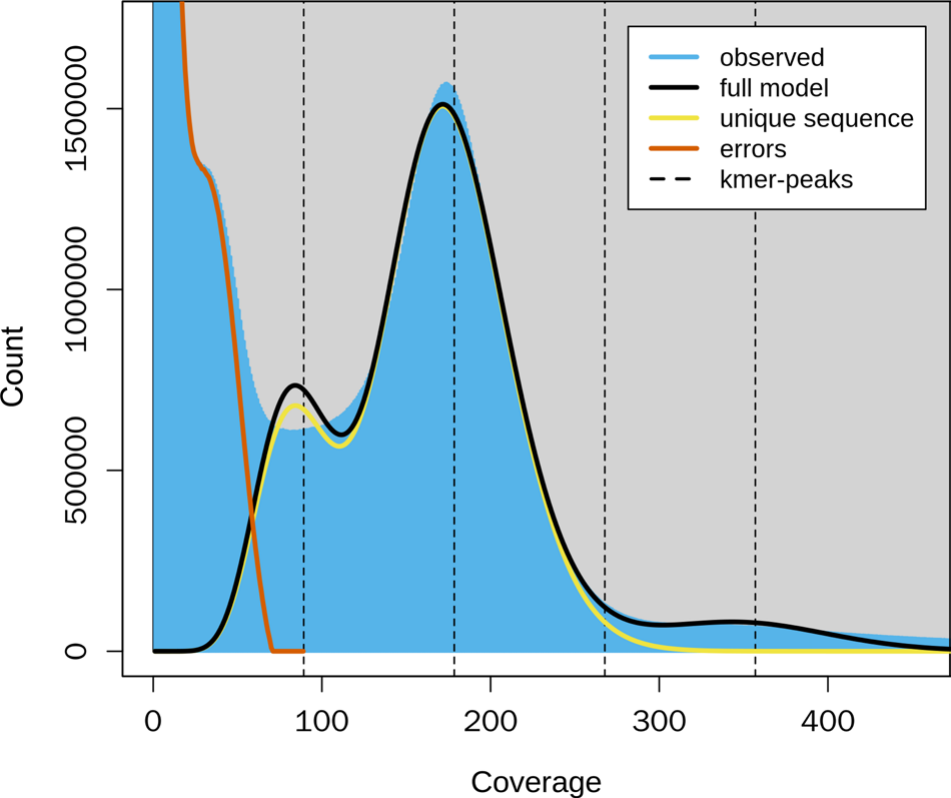
Genomic characteristics of *Megalurothrips usitatus* based on Illumina short-read data obtained in GenomeScope version 2.0 with 17 K-mer. The K-mer distributions showed double peaks: the first peak with a coverage of 100 indicates genome duplication and the highest peak with a coverage of 200 represents a genome-size peak. Genome size was calculated to be 255.81 Mb with a heterozygous rate of 0.85%.

Genomic characteristics were determined based on 55.86 Gb of short-read data using a K-mer-based statistical analysis in JELLYFISH version 2.1.3^15^ with the following parameters: ‘count -m 17 -C -c 7 -s 1 G -F 2’. Genome heterozygosity and genome size were estimated in GenomeScope version 2.0^16^ with default parameters. Based on 17-mer depth analysis, genome size and heterozygosity were estimated to be 255.81 Mb and 0.85%, respectively (Fig. 1).

### Genome assembly

We assembled a draft genome using wtdbg2 version 2.5 with default parameters^17^. We then had it polished using RACON version 1.4.13^18^ with parameters ‘-m 8 -x −6 -g −8 -w 500 -u’ and Pilon version 1.14^19^ with default parameters based on 98.30 Gb long reads and 55.86 Gb short reads.

**Fig. 2:**
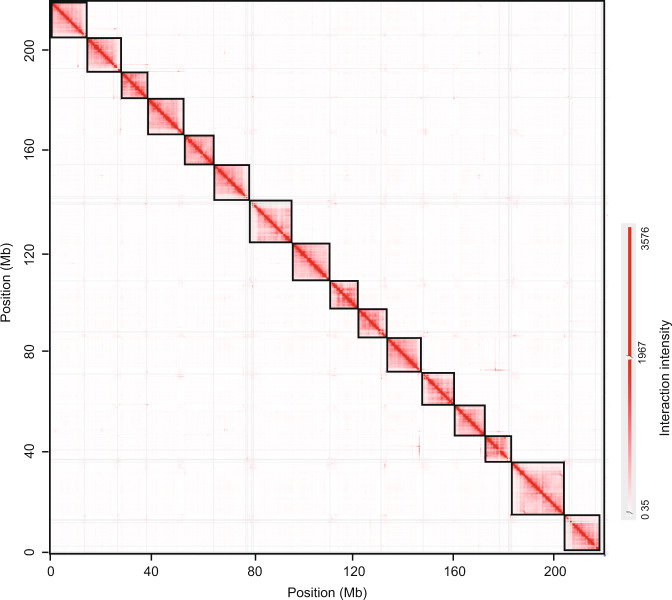
Genome-wide contact matrix of *Megalurothrips usitatus* generated using Hi-C data. Each black square represents a pseudo-chromosome. The color bar indicates the interaction intensity of Hi-C contacts.

A scaffolding pipeline based on Durand (2016)^20^ was used to generate a high-quality chromosome-scale genome. Initially, Hi-C data were mapped to the contig assembly using BWA-MEM version 0.7.17^21^ with the following parameters: ‘mem -SP5M’. Next, the DpnII sites were generated using the ‘generate_site_positions.py’ script in Juicer version 1.5^20^. The 3D-DNA pipeline (-r 2) was subsequently employed to order, orient, and cluster the contig^22^. After viewing Hi-C contact maps, the chromosome-scale genome was assembled in Juicebox version 1.11.08 (https://github.com/aidenlab/Juicebox). The genome assembly was screened for contaminant sequences by using the “Contamination in Sequence Databases” in NCBI. A total of 33 sequences were labeled as contaminant and removed (available in Figshare). To identify the mitochondrial genome, we amplified the cytochrome oxidase subunit 1 (COI) gene fragment with primer pairs LCO1490 and HCO2198, and obtained a DNA barcode sequence of approximately 610 bp^23^. We then used BLAST version 2.2.28^24^ (-evalue 1e-5) to find assembly sequences of a high similarity to the COI fragment (>98%), and identified one unplaced sequence (scaffold46) as mitochondrial sequence. The resulting chromosome-level genome was 238.14 Mb with a scaffold N50 of 13.85 Mb, maximum length of 20.88 Mb, and GC rate of 55.90% (Table 2). 91.89% of the genome was anchored to 16 pseudo-chromosomes (Table 2), which were well-distinguished from each other based on the chromatin interaction heatmap (Fig. 2).

**Table 2:** Statistics for the chromosomal-level genome of the *Megalurothrips usitatus*.

Features	Values
Total length (bp)	238,139,689
Longest scaffold length (bp)	20,884,914
Scaffold N50 (bp)	13,852,586
Scaffold N90 (bp)	10,644,695
GC (%)	55.90
Anchored to chromosome (Mb, %)	218.82 (91.89%)

### Predicting repeats

Repeat sequences were annotated in Extensive *de novo* TE Annotator (EDTA) version 1.9.4^25^. In brief, LTR retrotransposons were identified in LTR FINDER version 1.07^26^, LTRharvest^27^, and LTR retriever version 2.9.0^28^ with default parameters. Next, TIR Learner^29^ and HelitronScanner^30^ were used to classify DNA transposons with default parameters. RepeatMasker version 4.0.7 (-gff -xsmall -no_is)^31^ and RepeatProteinMasker version 4.0.7 (-engine wublast) were utilized to identify repeat sequences based on RepBase edition 20170127^32^. Repeats were masked with *de novo* predictions using RepeatModeler version 2.0.1 with parameters ‘-engine ncbi -pa 4’. Additionally, Tandem Repeats Finder^33^ was used to annotate tandem repeats with parameters ‘2 7 7 80 10 50 500 -f -d -m’. Overall, 20.20% of the assembled genome was classified as repetitive sequences in the *M. usitatus* genome (Table 3). Tandem repeat elements were found to be the most abundant (8.42%), followed by the terminal inverted repeat category (5.39%) (Table 3).

**Table 3:** Classification of repeat annotation in the *Megalurothrips usitatus* genome.

Class	Count	Masked length (bp)	Percent (%)
**LTR-retrotransposon**			4.28
Copia	2,408	1,536,255	0.65
Gypsy	7,985	5,493,251	2.31
Unknown	8,220	3,156,422	1.33
**Terminal inverted repeat**			5.39
CACTA	13,022	3,821,143	1.60
Mutator	15,562	5,594,823	2.35
PIF/Harbinger	264	117,748	0.05
Tcl/Mariner	78	47,698	0.02
hAT	10,259	3,260,929	1.37
**Non-terminal inverted repeat**			2.11
Helitron	15,366	5,027,332	2.11
**Tandem repeat**	239,289	20,056,514	8.42
**Total**	73,164	28,055,601	20.20

### Gene and functional predictions

Genes in the assembled genome were predicted using a combination of homology-based, transcriptome-based, and *ab initio* methods. Homology-based predictions involved downloaded sequences of peptides and transcripts from *Aptinothrips rufus* (http://v2.insect-genome.com/Organism/87), *Frankliniella occidentalis* (https://ftp.ncbi.nlm.nih.gov/genomes/all/GCF/000/697/945/GCF_000697945.3_Focc_3.1), and *Thrips palmi* (https://ftp.ncbi.nlm.nih.gov/genomes/all/GCF/012/932/325/GCF_012932325.1_TpBJ-2018v1). The IsoSeq version 3.4.0 workflow was utilized to generate 28,608 high-quality transcripts from the full-length transcriptome data, with quality parameters of 0.99 (https://github.com/PacificBiosciences/IsoSeq). Next, RNA-seq short data were mapped to the reference genome using HISAT2 version 2.2.1^34^ with the parameter ‘-k 2’. The mapped reads were then assembled into transcripts using StringTie version 2.4.0^35^ with default parameters. Homologous proteins and transcripts were aligned using Exonerate version 2.4.0 with default parameters to train the gene sets. Meanwhile, a sorted and mapped bam file of RNA-seq data was transferred to a hints file using the bam2hints program in AUGUSTUS version 3.2.3^36^ with the parameter ‘–intronsonly’. The trained gene sets and hint files were combined as inputs for AUGUSTUS version 3.2.3^36^, which predicted coding genes from the assembled genome with default parameters. Finally, homology-based, *de novo*-derived, and transcript genes were merged in MAKER version 2.31.10 to generate a high-confidence gene set^37^. It resulted in the annotation of 14,000 *M. usitatus* genes. The average transcript length was 2,243.30 bp with an average length of coding sequence (CDS) of 1,588.94 bp. The average exon number per gene was 7.38, and the average exon length was 303.85 bp (Table 4).

**Table 4:** Gene annotation statistics of the *Megalurothrips usitatus* genome.

Features	Results
Number of genes	14,000
Average gene length (bp)	4,612.39
Number of mRNAs	13,474
Average mRNA length (bp)	2,243.30
Average mRNA count per gene	1.10
Average CDS length (bp)	1,588.94
Average protein sequence length (bp)	529.65
Average exon length (bp)	303.85
Average exon count per gene	7.38

Gene structure and annotations were determined through several methods, including eggnog-mapper^38^ (-m diamond–tax_scope auto–go_evidence experimental–target_orthologs all–seed_ortholog_evalue 0.001–seed_ortholog_score 60–query-cover 20–subject-cover 0 –override), InterProscan version 5.0^39^ (-iprlookup -goterms -appl Pfam -f TSV), BLAST version 2.2.28^24^ (-evalue 1e-5), and HMMER version 3.3.2^40^ (–noali–cut_ga Pfam-A.hmm). These methods were used to search against multiple public databases, including NCBI non-redundant protein (Nr), Gene Ontology (GO), Clusters of Orthologous Groups of Proteins (COG), Kyoto Encyclopedia of Genes and Genomes (KEGG), Swiss-Prot, and Pfam. Most genes (91.74%) were successfully annotated with at least one public database (Table 5).

**Table 5:** Functional annotation of the *Megalurothrips usitatus* genome.

Database name	Annotated number	Percent (%)
NR	12,804	91.46
Swissport	10,026	71.61
GO	4,334	30.96
KEGG	6,891	49.22
COG	10,253	73.24
eggnog	10,253	73.24
Pfam	9,909	70.78
Bm	10,446	74.61
Dm	9,908	70.77
Total	12,843	91.74

Databases Bm and Dm were locally built in BLAST version 2.2.28^24^ using publicly available sequences of *Bombyx mori*^69^ and *Drosophila melanogaster*^70^, respectively.

### Comparative genomic analysis

To identify single-copy orthologous genes, we utilized the longest protein sequence of each gene from *M. usitatus* and multiple other species (Table 6), including *F. occidentalis*^41^, *T. palmi*^42^, *Acyrthosiphon pisum*^43^, *Triatoma rubrofasciata*^44^, *Columbicola columbae*^45^, *Aedes aegypti*^46^, *Danaus plexippus*^47^, *Tribolium castaneum*^48^, *Apis mellifera*^49^ and *Daphnia galeata*^50^. We performed all-to-all single-copy ortholog BLAST comparisons in OrthoFinder version 2.5.4^51^ with the parameters ‘-a blast -M msa’. We aligned the resulting single-copy orthologous genes using MAFFT version 7.487 (–auto)^52^ and further trimmed the poorly aligned regions using Gblocks version 0.91b^53^ (-t = p -b4 = 5). We maintained the genes that met the stationary, reversible and homogeneous (SRH) assumptions^54^ using IQ-TREE version 2.2.0^55^ with a p-value cut-off of 0.05. We finally obtained 1,573 single-copy genes under these criteria. Next, We used FASconCAT-G version 1.05.1^56^ to concatenate the genes to form a supermatrix, which was used for subsequent phylogenetic analysis.

**Table 6:** Genome datasets were used for comparative genomic analysis in the study.

Order	Family	Species	Database	Accession number	Reference
Thysanoptera	Thripidae	*Megalurothrips usitatus*		In this study	
Thysanoptera	Thripidae	*Frankliniella occidentalis*	NCBI	GCA_000697945.5	^41^
Thysanoptera	Thripidae	*Thrips palmi*	NCBI	GCA_012932325.1	^42^
Hemiptera	Reduviidae	*Triatoma rubrofasciata*	GigaDB	100614	^44^
Hemiptera	Aphididae	*Acyrthosiphon pisum*	NCBI	GCA_005508785.2	^43^
Phthiraptera	Philopteridae	*Columbicola columbae*	InsectBase	IBG_00199	^45^
Diptera	Culicidae	*Aedes aegypti*	NCBI	GCA_002204515. 1	^46^
Lepidoptera	Nymphalidae	*Danaus plexippus*	NCBI	GCA_018135715.1	^47^
Coleoptera	Tenebrionidae	*Tribolium castaneum*	NCBI	GCA_000002335.3	^48^
Hymenoptera	Apidae	*Apis mellifera*	NCBI	GCA_003254395.2	^49^
Anomopoda	Daphniidae	*Daphnia galeata*	NCBI	GCA_918697745.1	^50^

We performed a maximum likelihood analysis of concatenated sequences in IQ-TREE version 2.2.0^55^ with 1,000 UFBoot replicates (–bb 1,000 –model JTT + I + G4). The minimum correlation coefficient for the convergence criterion was set at 0.99 (-bcor 0.99). The age of each node was estimated using a correlated rates clock in MCMCTREE of PAML version 4.4^57^. To estimate the divergence times, we selected fossil records listed in Table 7.

**Table 7:** Fossils were used for estimating divergence times and calibration point prior settings in the analysis.

Node assigned	Fossils	Age (Ma)		Remarks
		Min	Max	
*Daphnia galeata* + Insecta		456	531	This calibration was based on the conclusion of (Rehm *et al*., 2011), which determined the divergence between Crustacea and Hexapoda ~510 Mya^71^.
Thysanoptera + Hemiptera + *Columicola columbae*		333	378	This calibration was based on the conclusion of (wang *et al*., 2016), which determined that divergence between that Psocodea and Condylognatha occurred around the Devonian and Carboniferous boundary ~357 Ma (378–333 Ma)^72^.
*Tribolium castaneum* + (*Danaus plexippus* + *Aedes aegypti*)	*Gallia alsatica* (Diptera: Rhagionidae)	242		Diptera was determined based on records of immature Diptera from the Triassic period (~242 Ma)^73^.
	*Moravocoleus permianus* (Coleoptera: Tshekardocoleidae)		293	This calibration was based on the oldest Palaeozoic beetles described from Sakmarian (290–293 Ma)^74^.
*Triatoma rubrofasciata* + *Acyrthosiphon pisum*	*Paraknightia magnifica* (Hemiptera: Paraknightiidae)	241		This calibration was based on the oldest described fossils of Heteroptera (~241 Ma)^75^
	*Aviorrhyncha magnifica* (Hemiptera: Aviorrhynchidae)		307	The oldest described fossils of Sternorrhyncha, are estimated to be from around 307 Ma^76^.
(*Frankliniella occidentalis* + *Megalurothrips usitatus*) + *Thrips palmi*		70	119	This calibration was based on the findings of (Johnson *et al*., 2018), which determined that the *Frankliniella* and *Thrips* diverged at 90 Ma (70–119 Ma)^77^.

**Fig. 3:**
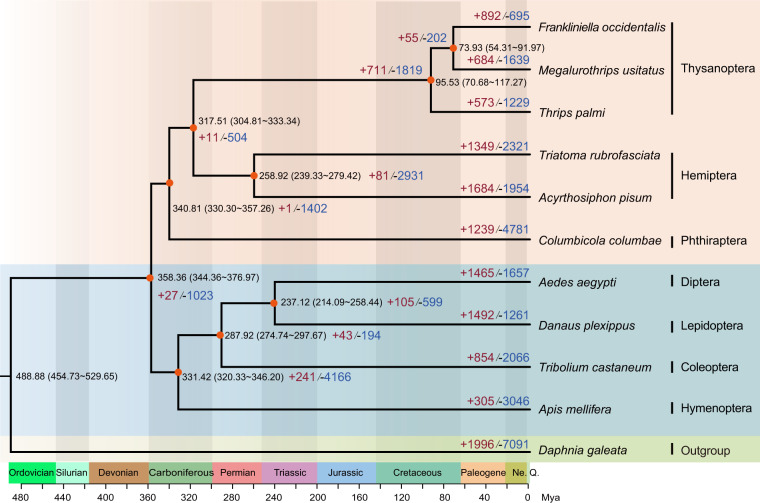
Genome evolution of *Megalurothrips usitatus*. A time-calibrated phylogenetic tree inferred from 1,573 single-copy orthologs using IQ-TREE version 2.2.0 was shown. The upper panel in wheat represents Paraneoptera insects and the lower panel in light-blue represents Holometabola. The divergence between *M. usitatus* and *F. occidentalis* diverged 73.93 Mya (Million years ago). Bootstrap support values based on 1,000 replicates are equal to 100 (orange dot). The number of expanded (+red) and contracted (−blue) gene families are shown for each lineage.

**Fig. 4:**
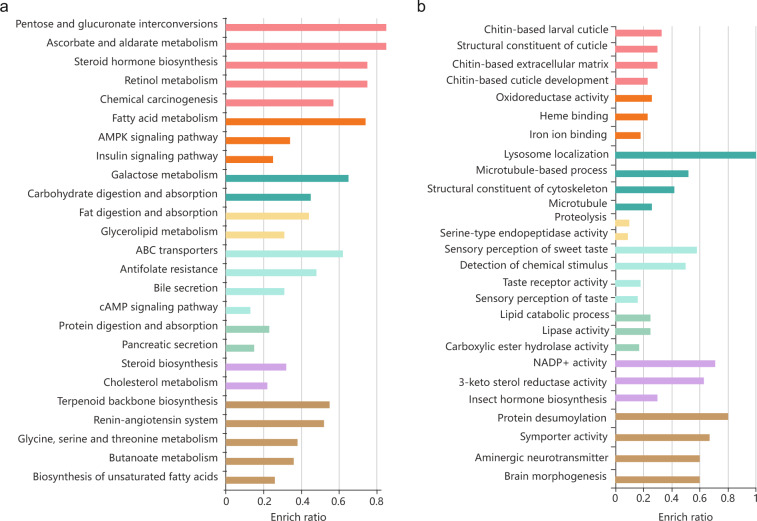
Functional annotation of expanded and contracted gene families. (**a**) Expanded genes. (**b**) Contracted genes. Each row represents an enriched function, and the bar length represents the enrichment ratio (input gene number/background gene number). Bar colors represent different clusters. If any cluster has more than five terms, the top five with the highest enrichment ratio are displayed.

Gene-family expansion and contraction were estimated using CAFÉ version 4.2 with parameters ‘lambda -s -t’, based on maximum likelihood and reduction methods^58^. Phylogenetic tree topology and branch lengths were considered when inferring the significance of changes to gene-family size in each branch. The results revealed 684 expanded gene families and 1,639 contracted gene families in *M. usitatus* (Fig. 3). Next, functional enrichment analysis (GO enrichment and KEGG pathway) was performed in KOBAS version 3.0^59^. Significantly enriched GO terms were those with an adjusted p < 0.05 under Fisher’s exact test. Expanded gene families were enriched in cAMP signaling pathway, fatty acid metabolism, detoxification metabolism (ABC transporters) and the ionotropic glutamate receptor pathway (Fig. 4a, available in Figshare). Contracted gene families were enriched in chitin-based cuticle development, sensory perception of taste and NADP + activity (Fig. 4b, available in Figshare).

## Data Records

Genomic PacBio sequencing data were deposited in the Sequence Read Archive at NCBI under accession number SRR22137485^60^.

Genomic Illumina sequencing data were deposited in the Sequence Read Archive at NCBI under accession SRR22137482^61^.

RNA-seq data were deposited in the Sequence Read Archive at NCBI under accession number SRR22137484^62^.

Full-length transcript isomer sequencing data were deposited in the Sequence Read Archive at NCBI under accession number SRR22137483^63^.

Hi-C sequencing data were deposited in the Sequence Read Archive at NCBI under accession number SRR22137481^64^.

The final chromosome assembly was deposited in GenBank at NCBI under accession number JAPTSV000000000^65^.

The contaminant file, single-copy orthologous genes, gene-family expansion and contraction, gene function annotation, and repeat annotation are available in Figshare^66^.

## Technical Validation

### DNA integrity

The integrity of extracted genomic DNA was determined using 0.75% agarose gel electrophoresis and analyzed with an Agilent 2100 Bioanalyzer (Agilent Technologies, USA). DNA concentration was measured using a Nanodrop 2000 spectrophotometer (Thermo Fisher Scientific, USA) and Qubit 2.0 (Thermo Fisher Scientific, USA). Absorbance at 260/280 nm was approximately 1.8.

### Assessment of genome assemblies

We assessed the accuracy of the final genome assembly by mapping Illumina short reads to the *M. usitatus* genome with BWA-MEM version 0.7.17^21^. The analysis showed that 96.52% of short reads were successfully mapped to the *M. usitatus* genome (Table 8). We further assessed the base quality of genome assembly by estimating the quality value score (QVS) using Merqury version 1.1^67^, which showed a high QVS of 32.65 (Table 8). These findings indicate that the quality of our assembled genome is high.

**Table 8:** Assessment metrics for the final genome assembly of *Megalurothrips usitatus*.

	Types	Results
Genome completeness	Complete BUSCOs (C)	97.40%
	Complete and single-copy BUSCOs (S)	97.00%
	Complete and duplicated BUSCOs (D)	0.40%
	Fragmented BUSCOs (F)	0.60%
	Missing BUSCOs (M)	2.00%
Genome accuracy	Mapping short-reads rate	96.52%
	Quality value scores (QVs)	32.65

Furthermore, we evaluated the completeness of the final genome assembly using Benchmarking Universal Single-Copy Orthologs (BUSCO version 3.0.2) insecta_odb10^68^, which includes 1,367 orthologous genes. The analysis revealed a high completeness of 97.40% for the *M. usitatus* genome with only 0.60% of BUSCO genes being fragmented, 2.00% being missing, and 0.40% being duplicated (Table 8). These BUSCO results were comparable to the completeness for other thrips genomes, such as *T. palmi* (97.20%), *F. occidentalis* (98.50%), and *A. rufus* (95.00%) (Table 9).

**Table 9:** Comparisons of genome assemblies of different thrips.

Species	Assembly level	Genome size (Mb)	Scaffold N50 (Kb)	BUSCO (%)	GC (%)
*Megalurothrips usitatus*	Chromosome	238.14	13,852	97.40	55.90
*Thrips palmi*	Chromosome	237.85	14,670	97.20	53.90
*Frankliniella occidentalis*	Scaffold	274.99	4,180	98.50	48.40
*Aptinothrips rufus*	Contig	339.92	5	95.00	48.60

## Data Availability

No specific codes or scripts were used in this study. All software used is in the public domain, with parameters clearly described in the Methods section.
